# Using newly linked hospital and population data to identify opportunities to reduce harms from sedative-hypnotic prescribing at population scale

**DOI:** 10.23889/ijpds.v9i5.2669

**Published:** 2024-09-18

**Authors:** Surain B Roberts, Mohammad A Imrit, Marwa F Ismail, Trong Shen, Christine Soong, Barbara Liu, Denise YF Mak, Vladyslav Kushnir, Laura C Rosella, Fahad Razak, Amol A Verma

**Affiliations:** 1 Li Ka Shing Knowledge Institute, Unity Health Toronto; 2 Institute of Health Policy, Management and Evaluation, University of Toronto; 3 Division of General Internal Medicine and Hospital Medicine, Sinai Health System; 4 Centre for Quality and Patient Safety, University of Toronto; 5 Division of Geriatric Medicine, Sunnybrook Health Sciences Centre; 6 Department of Medicine, University of Toronto; 7 Dalla Lana School of Public Health, University of Toronto; 8 ICES; 9 Institute for Better Health, Trillium Health Partners; 10 Temerty Faculty of Medicine, University of Toronto; 11 Department of Medicine, St Michael's Hospital

## Objective and Approach

Sedative-hypnotic medications are associated with harms such as delirium and falls, and may be overprescribed in hospitals. Measuring potentially inappropriate in-hospital sedative-hypnotic prescriptions requires distinguishing new initiations from pre-hospital prescriptions, but pre-hospital medications may not be captured in hospital data. To quantify physician- and hospital-level variation in sedative-hypnotic prescribing, we use linked data between GEMINI (which includes EMR data from >1.8 million admissions across 30 hospitals, approximately 60% of medical inpatient beds in Ontario) and ICES (which houses administrative healthcare databases, including outpatient prescriptions for adults ≥65 years). We will validate whether data in hospital clinical notes or pharmacy records can reliably identify pre-hospital prescriptions.

## Results

Using provincial health insurance number, 98.1% of GEMINI records could successfully be linked deterministically to ICES. Our exploratory sedative-hypnotic cohort included 90,806 general medicine patients treated by 437 physicians from 17 hospitals across Ontario from 2021-2022. Median age was 72 years and 48.7% were female. The proportion of patients receiving a new sedative-hypnotic prescription varied markedly across hospitals (median 0.21, range 0.04-0.41) and across physicians within hospitals (median within-hospital difference between lowest and highest prescriber: 0.18, range of within-hospital differences 0.07-0.67). Validation of new rather than continued prescriptions is underway. Full results will be ready for presentation at the conference.

## Conclusion

Sedative-hypnotic prescribing is common and highly variable in hospitals across Ontario, indicating an opportunity to improve patient safety.

## Implications

Based on these analyses, the province of Ontario has made reducing in-hospital sedative-hypnotic prescribing a provincial priority for 2023-2024.

